# Development and validation of a sliding type continuous passive motion automation device for evaluation and rehabilitation of frozen shoulder: a pilot study

**DOI:** 10.3389/fresc.2025.1639249

**Published:** 2025-08-11

**Authors:** Jewoo Lee, Sung-sik Yun, Kyung Rok Oh, Sun Gun Chung, Wonjae Hwang, Keewon Kim, Kyu-jin Cho

**Affiliations:** 1 Department of Mechanical Engineering, Seoul National University, Seoul, Republic of Korea; 2 Department of Rehabilitation Medicine, Seoul National University Hospital, Seoul, Republic of Korea; 3 Department of Rehabilitation Medicine, Seoul National University College of Medicine, Seoul, Republic of Korea

**Keywords:** frozen shoulder, rehabilitation, continuous passive motion (CPM), joint stiffness, robot

## Abstract

**Background:**

Frozen shoulder (FS) is a condition that results in pain and restricted range of motion (ROM) in the shoulder joint, impacting daily activities. Current rehabilitation methods, including physical therapy and passive range of motion (PROM) exercise, can be limited by cost and availability. This study aimed to develop and test a novel robotic CPM device capable of measuring joint stiffness and improving accessibility and self-exercise effectiveness for FS patients.

**Methods:**

A 3-armed randomized clinical trial was conducted with 12 FS patients allocated into three groups: (1) hot pack treatment (negative control), (2) PROM by physiotherapists (active control), and (3) robotic CPM device-assisted exercise (intervention). ROM, pain levels using the Visual Analogue Scale (VAS), and the Shoulder Pain and Disability Index (SPADI) were measured at baseline, immediately post-intervention, and after a 6-week follow-up. A linear mixed model was applied for inter-group and intra-group analyses. Torque and stiffness were calculated using sensor data collected by the robotic device and Inertial Measurement Units (IMUs) for precise monitoring.

**Results:**

The intervention group showed significant improvement in ROM compared to the negative control group, particularly in external rotation (p = 0.022). Intra-group analysis for the intervention group revealed average ROM increases of $14.52^{\circ }$, $16.72^{\circ }$, and $14.19^{\circ }$ for flexion, abduction, and external rotation, respectively. Passive stiffness in the intervention group significantly decreased in abduction (p = 0.010) and external rotation (p < 0.001). Pain levels and SPADI scores decreased across all groups, with no statistically significant differences noted between the intervention and the postive control groups.

**Conclusions:**

The developed robotic CPM device demonstrated potential in improving ROM and reducing passive stiffness in FS patients, showing comparable results to therapist-assisted exercise. While the device enhances accessibility and self-monitoring capabilities, further studies are required to validate its use in home settings and assess its impact on long-term motivation for self-exercise.

**Clinical Trial Registration:**

The clinical trial was approved and registered under Seoul National University Hospital Institutional Review Board (IRB No. 2206-161-1335).

## 1 Introduction

The shoulder is a ball-and-socket joint, which enables the widest range of motion (ROM) and highest degrees of freedom (DOF) among the other joints. Due to the versatility, the shoulder joint is crucial for activities of daily living (ADL) such as lifting, reaching and throwing. Four joints (glenohumeral, acromioclavicular, sternoclavicular and scapulothoracic) with corresponding tendons, ligaments and muscles provide static and dynamic stability during the complex shoulder movements (1). Any single disruption on the components of shoulder structure can result in pain and instability of shoulder.

Adhesive capsulitis, also known as frozen shoulder (FS), is a condition that causes pain and stiffness in the shoulder joint, resulting in a loss of ROM (2). FS can be characterized by the thickened tissues surrounding shoulder joints, or the shoulder capsules. FS is estimated to affect 2% of the general population at minimum, with the incidence peak at the age of mid-50s (3, 4). Although the exact cause which thickens the shoulder capsules and reduces the joint volume is not clarified, FS is linked with various comorbidities, including stroke (5) and diabetes (6).

Despite the belief that FS is a self-healing disease, 20 ∼ 50% of patients suffer from persistent shoulder stiffness and pain as a chronic condition (4). Several treatment options including physical therapy, medications, heat and cold therapy, injections or surgeries are offered to the patients to improve such symptoms and increase the chances of a full recovery. Among the various options, rehabilitation exercise should always be included in the treatment of FS, in order to achieve or maintain the shoulder functionality (2).

Among various exercises, passive range of motion (PROM) exercise is considered to be one of the most effective methods (7, 8). Without the participants’ active control of limbs, PROM moves the shoulder joint to the end range, so that collagen fibers comprising the shoulder joint are elongated and help improving ROM (9). However, it’s crucial to note that during PROM, as the joint reaches its end range, a significant reaction force is generated, which can pose safety concerns (10). Therefore, it is imperative to consider not only the position of the shoulder joint but also the joint stiffness. This underscores the necessity for physical therapists who can accurately measure joint stiffness and ensure the safe application of force throughout the full range of motion (11, 12). Although exercise with physical therapists can have higher satisfaction ratio than self-exercise method (13), intrarater and interrater reliability of diagnosis are significantly low according to therapists (14). Moreover, expensive price for the therapy session makes patients choose self-exercise methods which has advantages on cost-effectiveness (15). However, patients conducting self-exercise are not easy to verify effects of the exercise and need to be motivating themselves to continue, which often leads to failure. Tanaka et al. (12) compared effectiveness of physical therapists on self-exercise, where 47% of patients in the self-exercise group were eventually classified as not-treated.

Robotic rehabilitation devices are gaining interest as a new alternative to reduce the cost burdens imposed on patients and help monitor the rehabilitation progress themselves. To deal with the wide ROM of the shoulder joint and misalignment issues, rigid-type rehabilitation robots apply multiple actuators (11). With torque and force sensors applied on the actuator and the limbs of the robots, rigid-type robots offer precise status-measuring functions. However, due to the high costs and bulky size, accessibility to the device is not easily accessible to ordinary patients (6, 12, 16). Moreover, due to complicated control methods, rigid robots are currently constrained to in-clinic usage (15). Additionally, rigid robots often require significant time investment for device customization to ensure proper joint alignment with the patient (17, 18). Soft-type robots solve these problems by using cable-driven or pneumatic actuators (15, 19) and improving wearing conformity. But non-linear behaviors of the soft materials hinder monitoring functions and cannot provide kinetic measurements, which is critical for joint stiffness calculation in FS diagnosis stage (20). Furthermore, the time required for wearing soft-type robots can be considerable, adding to their practical limitations (21, 22).

In this research, joint-stiffness measurable sliding type CPM automation device was developed to fill the gap between the human therapists and the current robotic devices by covering wide ROM with monitoring function for motivating self-exercise patients and still being manufactured with reasonable costs. By incorporating a force measurement function, the device provides real-time assessment of stretch tolerance during exercises, allowing for the monitoring of pain levels and shoulder functionality (23). This data enables healthcare professionals to tailor exercise plans based on individual stretch tolerance levels, optimizing therapeutic outcomes. The device covers a wide ROM with its cable-driven actuator, facilitating exercises in flexion, abduction and external rotation directions. With its simple mechanism and control methods, the device can easily be set up and manipulated within 5 minutes, which is sufficient for usability (24). Clinical trials with 12 FS patients were conducted to verify the therapeutic effect of the device.

## 2 Methods

### 2.1 Physical therapist analysis

Exercise methods of human therapists in CPM exercise were analyzed in prior to the design of the device. The exercise sessions were observed at a university hospital. The session usually took about 45 min, including 15 min of hot pack treatment prior to the session.

**Figure 1:**
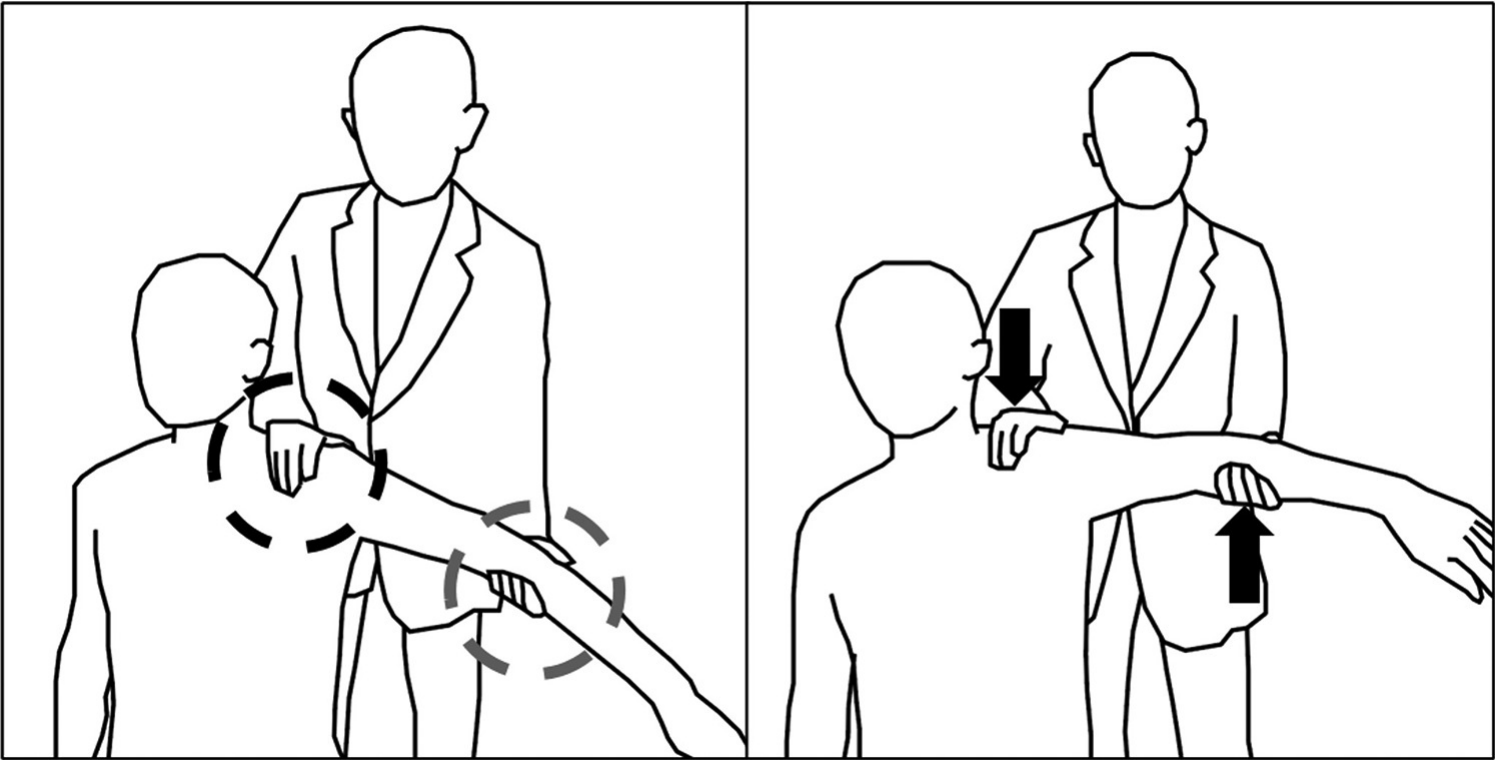
Exercise with a physical therapist: One hand senses shoulder stiffness while the other elevates the shoulder to the stiffened region.

Exercise principles of human therapists could be classified into three stages: sensing, actuation, and decision. First, sensing stages are conducted by putting hands on the patients. As can be seen from (Figure 1), human therapists put their hands on two spots of the patient, one on the trapezius muscle and the other on the arm being elevated. The hand on the trapezium detects whether the patient has proper scapulohumeral rhythm (25), while the hand on the arm checks the stiffness of the shoulder. Second, the actuation stage is mainly carried out by the hand on the moving arm with rotating the shoulder joint. Human therapists have the ability to move their hands freely to rotate shoulder joints in three main directions: forward flexion (FF), abduction (Abd), and external rotation (ER). The hand on the trapezium acts as preventing excessive movements of the scapula and maintaining the scapulohumeral rhythm. Third, in the decision stage, the therapist decides whether to move the joint further or not by integrating whole senses acquired from the patient.

Based on the human-therapist analysis, requirements for developing a robotic CPM device could be derived from both a hardware and software point of view. For the hardware, the device should provide passive motion in three different motions with the full ROM. For the software, the device should detect the end-range of the shoulder joint in each direction and determine when to release the passive motion.

### 2.2 Hardware design

**Figure 2:**
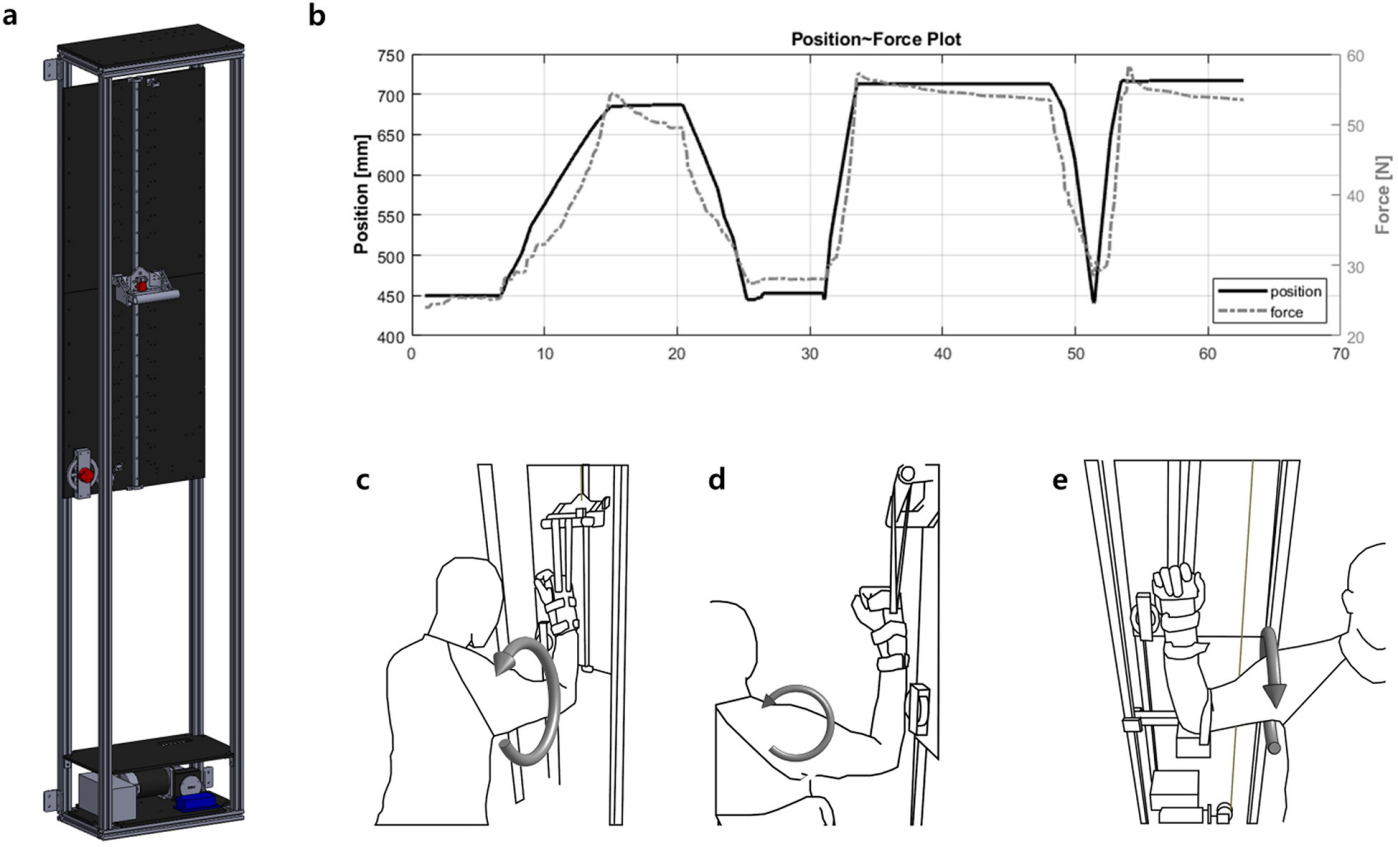
**(a)** Device modeling, **(b)** Operation protocol: After manually setting the optimal force threshold, the rehabilitation movement is performed 20 times, with a 10 s pause at the threshold to allow sufficient stretching, **(c)** Flexion, **(d)** Abduction, **(e)** External rotation.

The device (Figure 2) is an aluminum-frame-based, cable-driven system with a handle attached to a linear guide on which the arm of the patient is held using a wrist brace (Formfit®, Össur, Reykjavik, Iceland). The geared motor (9DCW24, DKM Motors Co. Ltd., Incheon, Korea) is mounted at the bottom of the device and moves the handle via cable-pulley system. The position of the handle is measured using a rotary encoder attached to the device. The rotary encoder and the motor were separated in order to measure the movement of the handle when the motor is not moving, which enabled easier position calibration.

When using the device, patients sit on a chair where a shoulder pad is attached. The pad covers the shoulder being lifted and prevents excessive elevation of the scapula, thereby controlling scapular compensation during passive shoulder movements. By adjusting the sitting position, patients can perform three degrees of freedom exercises. When conducting external rotation exercise (Figure 2e), an additional module is added to the device in order to fix the elbow of the patient and make a linear up-and-down motion of the handle into the rotating motion of the shoulder joint. The device operates without being constrained by the alignment of the elbow and shoulder joints. Additionally, the device does not require customization for joint alignment, even for patients of varying heights.

### 2.3 Sensing and control

The device utilizes two load cells (333FDX, Ktoyo Co. Ltd., Gyeonggi-do, South Korea) to mimic the sensing part of human therapists. The load cell embedded in the handle Figure 2 measures the vertical force during the CPM exercise and is further used for calculating torque applied to the shoulder joint. The other load cell attached to the shoulder pad measures the force exerted by the scapula and could be used to monitor the muscle synergy during shoulder movements.

The device is operated using a controller with a Teensy 3.2 board inside. Load cell data is accumulated using an Arduino board and transmitted to the controller through serial communication. Overall operation is monitored with a computer program developed with Processing, on which control parameters including force threshold, motor speed, and number of repetitions can be adjusted.

### 2.4 Inverse kinematics

During the rehabilitation exercises with the device, IMU data of the patients were collected for the reference data of the shoulder joint angle. We attached 5 IMU sensors (MTw Awinda, Xsens Inc., Culver City, USA) onto the limbs of the patient and 1 IMU sensor on the motor pulley to synchronize the movement of the human joint with the motor actuation.

To calculate the shoulder angle with the IMU data, the patient takes the initial calibration posture (standing tall with a straight back and arms relaxed at the sides) with one IMU attached on the back, two IMUs on the upper arm, and two IMUs on the lower arm. The directional vector of each segment is estimated using the rotation matrix. Shoulder angle during flexion and abduction exercise is calculated based on the rotation angle of the upper arm vector from the chest vector, while the rotation angle of the lower arm vector from the chest vector is used for the external rotation exercise.

Torque applied to the shoulder joint is calculated using the shoulder angle acquired from the IMU data, force measured by the device, statistically estimated anthropometric values, and additional distance measured on-site. In cases of flexion and abduction, the estimated torque is as follows:

$$\tau =F_{\mathrm{load}\;\mathrm{cell}}*\hat{l}-m_{\mathrm{eq}}*g*\hat{r}$$ (1)

where $\bar{l}$ is the horizontal distance of the shoulder joint from the device, $m_{\mathrm{eq}}$ is the equivalent mass of the whole arm, $\bar{r}$ is the horizontal distance of $m_{\mathrm{eq}}$ from the shoulder joint, and *g* is the gravitational acceleration constant.

Torque during the external rotation movement is estimated as follows: As the elbow support module is applied for the external rotation movement, elements used in the formula were modified. ${\hat{m}}_{\mathrm{eq}}$ is the equivalent mass of only the lower arm and hand; $\hat{r}$ is the horizontal distance of ${\hat{m}}_{\mathrm{eq}}$; l is the distance from the elbow to the strap; θ is the angle between the strap of the wrist brace and the device; and ϕ is the angle of the lower arm from the horizontal axis calculated from the IMU data.

$$\tau =\frac{F_{\mathrm{load}\;\mathrm{cell}}}{\cos\left(\theta \right)}*l*\cos\left(\theta -\phi \right)-{\hat{m}}_{\mathrm{eq}}*g*\hat{r}$$ (2)

**Figure 3:**
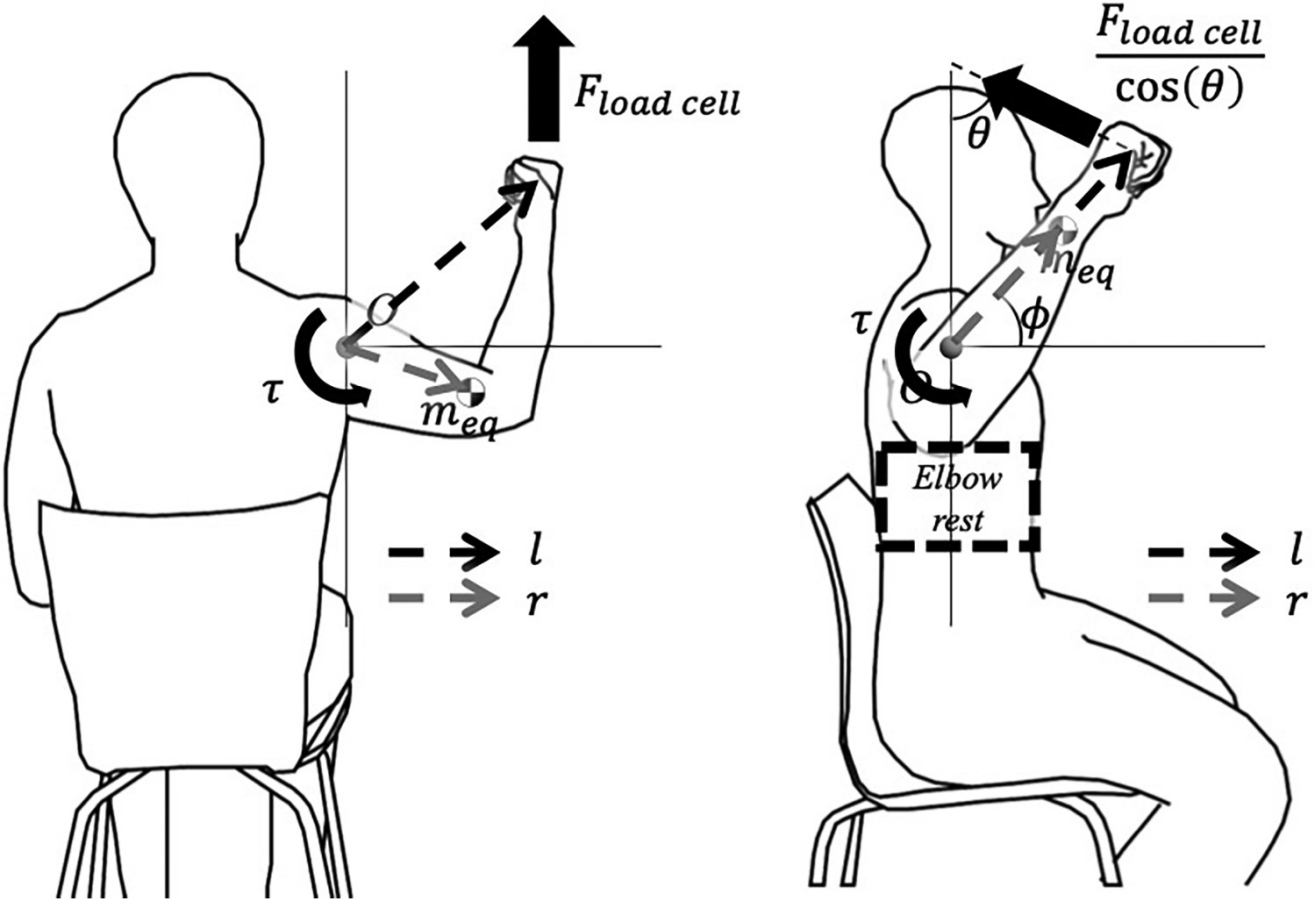
Free body diagram: **(a)** During flexion or abduction, **(b)** During external rotation. Variables are defined as l (distance from the origin to the wrist strap), r (distance of the lower arm’s equivalent mass from the origin), θ (angle between the wrist strap and the device), and ϕ (angle of the lower arm from the horizontal axis).

The torque Equations 1, 2 used in the calculation are illustrated in the free body diagram Figure 3.

### 2.5 Biomechanical parameters

At the first visit, patient demographics and baseline clinical data were collected, including age, height, weight, ROM in flexion, abduction, and external rotation, as well as VAS and SPADI scores. During each robotic exercise session in the intervention group, biomechanical data were acquired via IMU sensors and load cells embedded in the robotic device.

ROM was measured using IMU sensors attached to the upper and lower arms, and shoulder angles were derived from relative orientation changes. For flexion and external rotation, limited shoulder mobility due to device-chair distance was mitigated by placing a cushion on the participant’s lap to allow slight torso inclination and expand rotational freedom. Among the 20 repetitions performed per session, the last five were selected for analysis to minimize adaptation effects.

**Figure 4:**
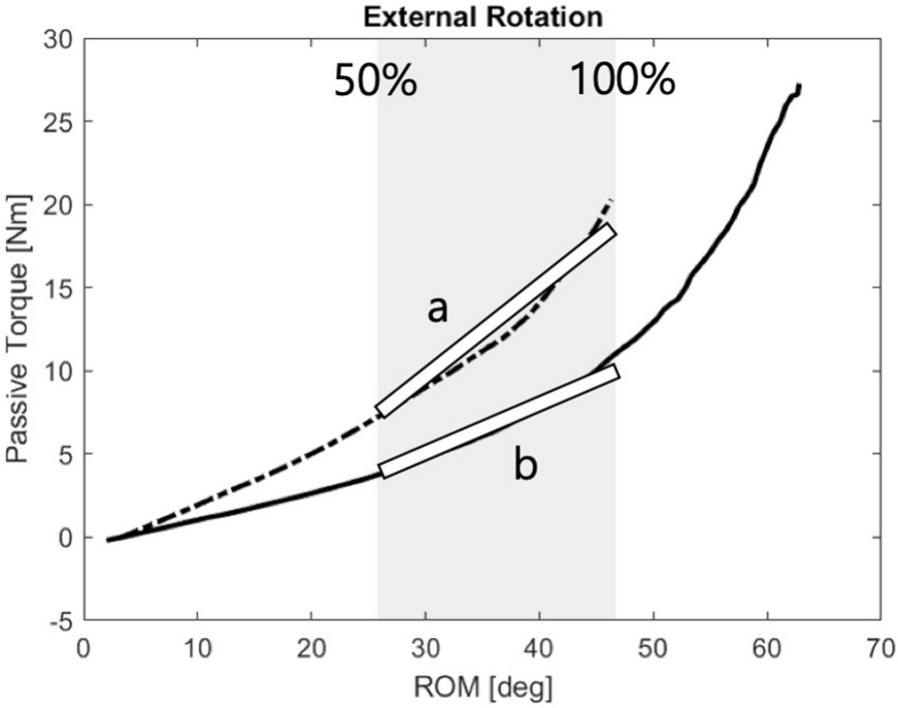
Passive stiffness calculation: Stiffness was assessed using a 1st-order fitting between 50% and 100% of the first visit ROM-passive torque relationship.

To quantify passive stiffness, torque-angle data were obtained by synchronizing load cell and IMU measurements. Stiffness was calculated by fitting a first-order linear regression to the torque-RoM curve between 50% and 100% of the motion range in each direction (26, 27). The resulting stiffness indices served as objective indicators of joint resistance and rehabilitation progress Figure 4.

## 3 Experimental setup

### 3.1 Participants

12 frozen shoulder patients from the Seoul National University Hospital were recruited Table 1. Patients diagnosed with adhesive capsulitis of shoulder or suffering from shoulder pain with decreased ROM for more than 3 months were invited. Exclusion criteria were those who has rotator cuff tear, glenohumeral osteoarthritis, systematic rheumatic disease, neurological diseases including strokes which affect shoulder ROM or those who had any kind of surgery on the affected side shoulder. All subjects provided informed consent to a protocol approved by Seoul National University Hospital Institutional Review Board (IRB No. 2206-161-1335).

**Table 1:** Patient characteristics.

Patient ID	Sex	Age (yr)	Symptom duration (m.)	Group	Initial assessment		
					FF	Abd	ER
Patient 1	F	63	6	Negative control	110	90	90
Patient 2	F	50	8	Negative control	150	90	50
Patient 3	F	61	5	Negative control	130	120	40
Patient 4	F	61	9	Active control	130	110	30
Patient 5	F	60	10	Active control	110	100	40
Patient 6	F	48	29	Active control	140	130	40
Patient 7	M	76	7	Active control	130	100	40
Patient 8	F	47	5	Intervention	140	100	40
Patient 9	M	81	3	Intervention	120	80	50
Patient 10	F	70	48	Intervention	150	140	40
Patient 11	F	50	8	Intervention	110	120	70
Patient 12	F	50	3	Intervention	130	70	30

### 3.2 Clinical trial design

This study was designed as an exploratory pilot trial to evaluate the feasibility, safety, and preliminary efficacy of the robotic CPM device in patients with frozen shoulder. A 3-armed clinical trial was conducted in which participants were randomly allocated to (1) hot pack treatment: negative control, (2) exercise with human therapists: active control, and (3) exercise with the robotic device: intervention group. The primary outcomes were range of motion (ROM) in flexion, abduction, and external rotation, as well as biomechanical measures such as passive resistive torque and joint stiffness. Secondary outcomes included pain and functionality of the affected shoulder, assessed using the Visual Analogue Scale (VAS) and the Shoulder Pain and Disability Index (SPADI).

**Figure 5:**
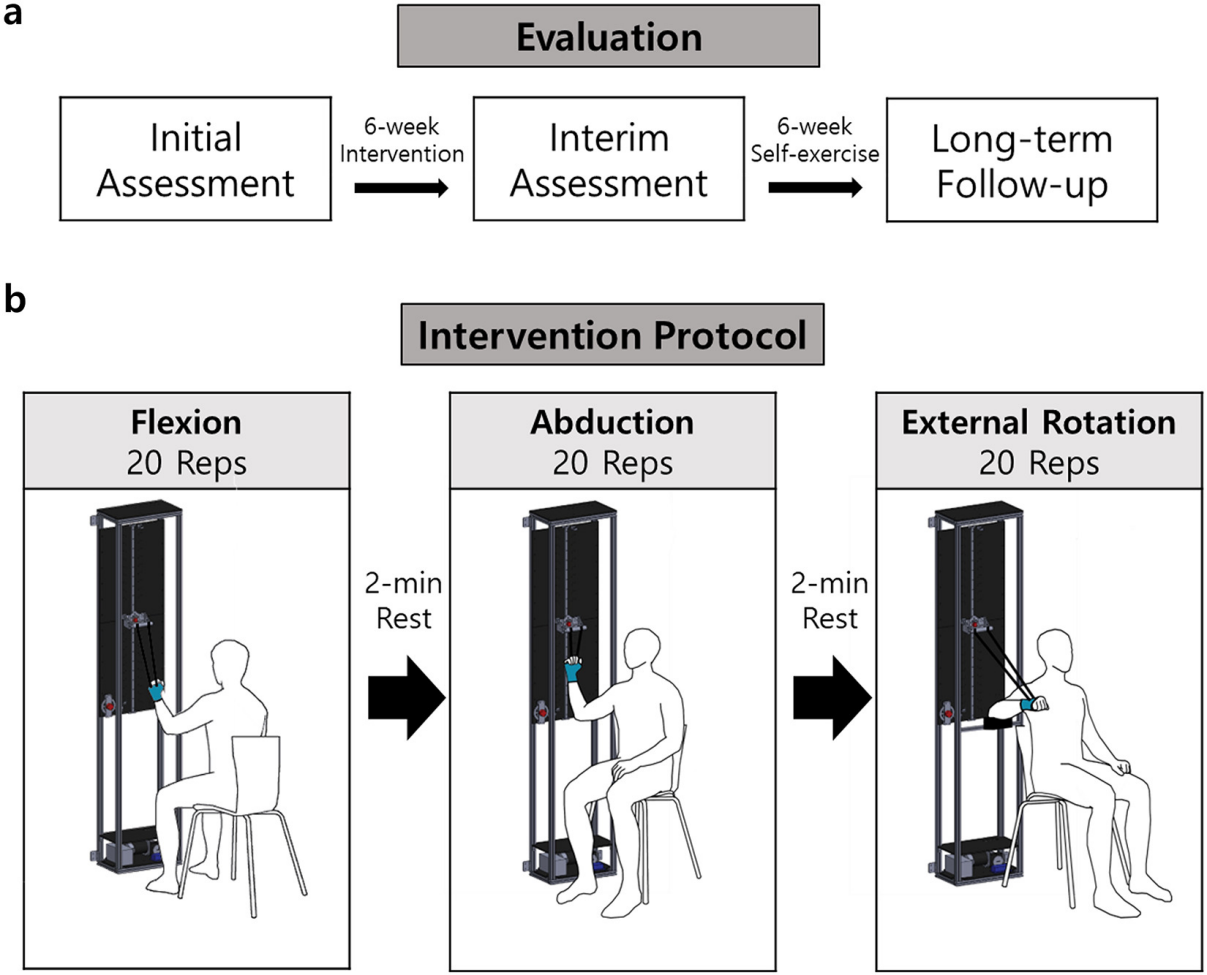
Evaluation and intervention protocol: **(a)** Evaluation is conducted three times over 12 weeks. **(b)** Intervention involves 20 repetitions in each direction.

The hypothesis of this study was that the intervention group would demonstrate therapeutic effects comparable to the active control group and superior to the negative control group. After allocating each patient into one of the three arms, baseline measurements were completed, followed by a 6-week intervention (Figure 5a). The second and third assessments were conducted immediately and six weeks after the intervention. Variations in outcome measures were used for quantitative comparisons between the three groups.

This study was not double-blinded. Due to the nature of the interventions, participants were aware of their assigned groups, and outcome assessments were performed by the treating therapists and researchers involved in device operation. While assessor blinding was not feasible, objective measures (e.g., ROM and torque collected via sensors and IMUs) were prioritized to minimize subjective bias. This limitation was considered in the trial design.

### 3.3 Experimental procedure

Participants allocated in negative control group received hot pack treatment for 20 min once a week. Negative control group was designed based on the current therapeutic protocol conducted by general university hospitals. Active control group participants received both the hot pack treatment followed by PROM exercise by therapists for 20 min a week. The exercise was comprised of flexion, abduction, and external rotation, 20 times each.

Participants in the intervention group received the hot pack treatment and robotic exercise therapy twice a week, which reflects easier accessibility to the robotic device compared with human therapists. For the patients in the intervention group, the overview of the measurement protocol is shown in (Figure 5b). The robotic device was positioned on a square grid sheet so that the sitting position of a participant is fixed for every visit. To minimize scapular compensation during shoulder movements, patients were seated with a shoulder pad designed to restrict excessive scapular elevation and promote proper scapulohumeral rhythm. After fixing the participant’s wrist on the wrist brace of the device, the handle was elevated with the controller until the patient felt high enough stretching strength while measuring the corresponding force with the robotic device so that the force threshold was set. After setting the force threshold, the handle was lowered to the initial position, where shoulder angles were set to $60^{\circ },45^{\circ }$, and $100^{\circ }$ for flexion, abduction, and external rotation direction. During the exercise, motor speed was fixed to manipulate the handle to move 0.15 m/s. While elevating the handle, once the force value on the handle exceeded the force threshold, the motor stopped for 10 s to ensure enough time for stress relaxation and then lowered to the initial position.

On the first visit, the patients in all groups were handed out with self-exercise manuals and encouraged to follow the guidelines but were not checked from then on.

### 3.4 Data analysis

Statistical analyses were conducted to evaluate both intra-group and inter-group rehabilitation effects. The primary outcomes were range of motion (ROM) in flexion, abduction, and external rotation, as well as biomechanical measures such as passive resistive torque and joint stiffness. Secondary outcomes included the Shoulder Pain and Disability Index (SPADI) and Visual Analog Scale (VAS) scores for pain.

For within-group analysis in the intervention group, a two-sample t-test (MATLAB R2023a, ttest2 function; MathWorks Inc., Natick, MA) was used to compare passive resistive torque between the first and last sessions.

To compare treatment effects across groups, a linear mixed model was applied to account for repeated measures at three time points: baseline, interim, and post-intervention. Fixed effects were defined as group, time, and baseline values; the random effect was participant identity. An interaction term between group and time was initially included but excluded from the final model due to lack of significance. All mixed model analyses were performed using SAS software (version 9.4; SAS Institute Inc., Cary, NC).

## 4 Result

### 4.1 Baseline measures

The participants were divided into three groups: negative control (3 participants), active control (4 participants), and the intervention group (5 participants), with average ages of 58.0, 61.3 and 59.6 years respectively Table 1. There were no statistically significant differences between the groups in terms of age, weight, duration of symptoms, or other relevant indicators. Similarly, the initial assessment showed no significant differences between the three groups.

### 4.2 Inter-group analysis of changes in ROM and SPADI test results

**Figure 6:**
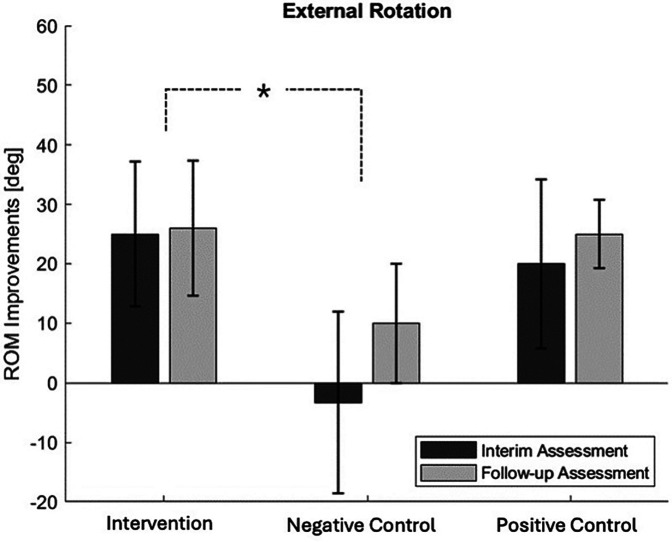
ROM improvement comparison: External rotation ROM improvement compared between groups.

When comparing the ROM evaluation results across the three groups Table 2, a gradual increase in all directions of ROM was observed as the treatment sessions progressed, except for abduction in the thermo-therapy group. However, there were differences in the degree of improvement between the groups, with the most notable difference seen in external rotation Figure 6. Compared to the negative control group, the intervention group showed a faster recovery in external rotation ROM (p-value = 0.022). The difference between the negative control and the intervention group became more pronounced during the interim assessment. Although the external rotation ROM in the negative control group showed some recovery during the follow-up assessment, the extent of improvement was still smaller compared to the intervention group. The SPADI test also showed a gradual decrease in pain levels, although statistical significance was not achieved (p-value = 0.642).

**Table 2:** Evaluation result comparison across groups.

Measure		Negative control			Active control			Intervention group		
		IA^a^	2nd^b^	3rd^c^	IA	2nd	3rd	IA	2nd	3rd
ROM [deg]	FF	130.00	140.00	160.00	127.50	150.00	145.00	126.67	139.17	151.67
	Abd	100.00	106.67	133.33	110.00	122.50	127.50	95.00	111.67	125.00
	ER	60.00	56.67	70.00	37.50	57.50	62.50	43.33	64.17	66.67
SPADI	Pain	66.00	34.00	32.67	67.50	43.50	25.00	56.67	35.33	30.33
	Disability	63.33	22.08	23.75	69.06	32.50	23.44	45.21	29.79	24.58
	Total	64.36	26.66	27.18	68.46	36.73	24.29	49.61	31.92	26.77

^a^IA: initial assessment.

^b^2nd: interim assessment.

^c^3rd: follow-up assessment.

### 4.3 Intra-group analysis of changes in ROM

**Figure 7:**
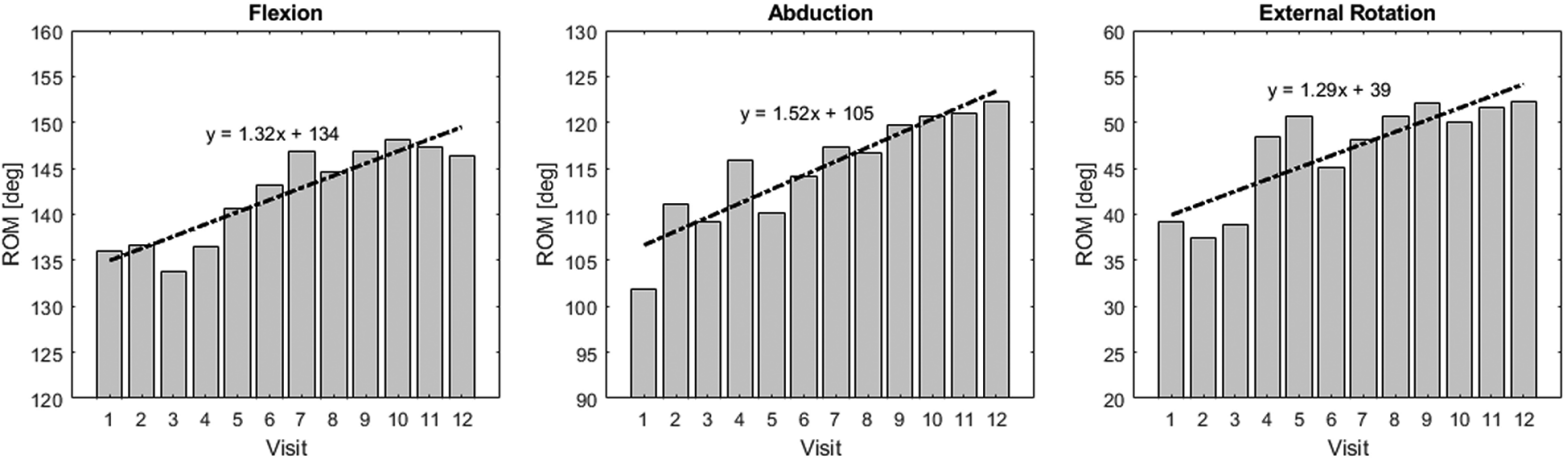
ROM improvement in the intervention group: Bar plot of ROM at each visit for the intervention group.

When examining the average ROM in each exercise direction of participants in the intervention group, a gradual increase in ROM was observed with each session Figure 7. Compared to the first session, by the 12th session, the ROM increased by an average of $14.52^{\circ }$ in the flexion, $16.72^{\circ }$ in the abduction, and $14.19^{\circ }$ in the external rotation. Since the ROM is determined by the force threshold set by the participant for the robot, it can be inferred that the participant’s stretch tolerance increased with each session.

### 4.4 Intra-group analysis of changes in passive stiffness

**Figure 8:**
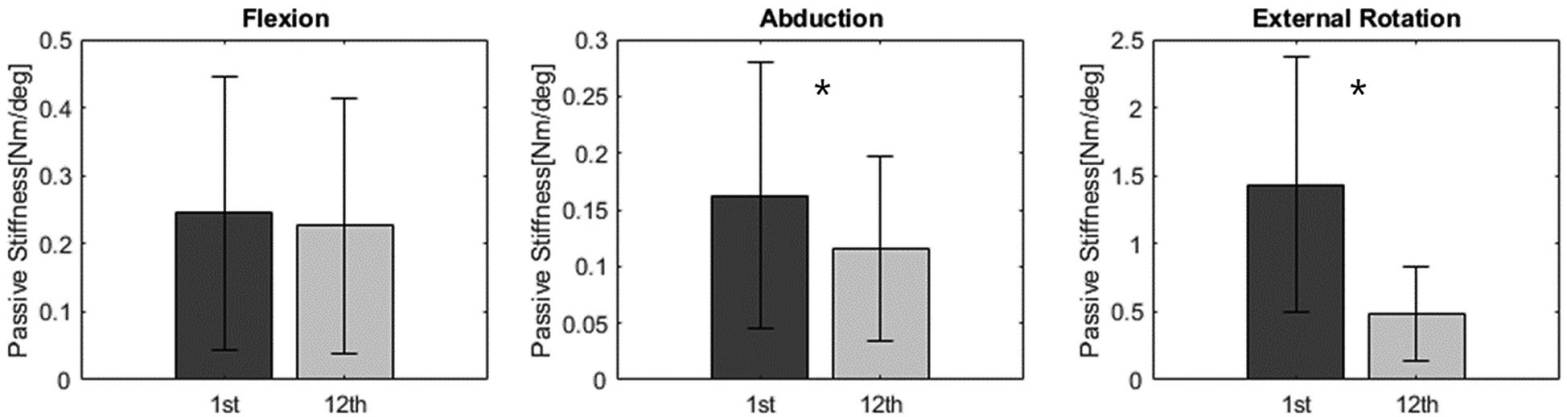
Passive stiffness result: Passive stiffness in the intervention group significantly decreased in abduction and external rotation.

Passive stiffness values in the intervention group significantly decreased from the first visit to the 12th (last) visit, particularly in abduction and external rotation Figure 8. Specifically, passive stiffness in abduction decreased from 0.17 to 0.10 (p-value = 0.010), and in external rotation, from 1.29 to 0.46 (p-value < 0.001). In flexion, passive stiffness showed a decrease from 0.27 to 0.21; however, this change was not statistically significant (p-value = 0.590).

## 5 Conclusion and discussion

A 3-armed clinical trial investigating the effectiveness of the developed robotic CPM device was conducted with 12 patients randomly allocated to each group. In comparison with the thermo-therapy-only treatment, the developed device showed greater ROM increases and SPADI decreases with statistical significance. There was no statistical significance in the comparison between the intervention group and the active control group, indicating that the robotic treatment demonstrated an effectiveness comparable to that of human therapist-assisted exercise.

The developed robotic device was designed to apply tangential forces in multiple directions to the shoulder joint using a single motor and cable mechanism. This design enhances its commercial viability and versatility, making it suitable for practical use in clinical settings. The device also features the capability to quantitatively measure joint stiffness while performing passive range of motion (PROM) exercises. This not only ensures safe application in treating FS patients, but also enables simultaneous measurement of stiffness and ROM, offering valuable insights into joint mechanics in frozen shoulder.

During FF and Abd direction exercises, human therapists rotate their actuating hand in an arc shape so that the upper arm of the affected side can naturally move around the shoulder socket as the center of the trajectory. In contrast, the developed device was intentionally designed to move the wrist along a linear trajectory, which allowed significant simplification of the actuator mechanism and enabled a more compact, low-profile design. Despite this linear path of the device, the shoulder joint itself traces an arc-like motion because the design utilizes the natural movement of the elbow joint to approximate the therapist’s arc trajectory. This approach effectively preserves the desired shoulder kinematics while offering mechanical and usability advantages. However, it requires normal elbow mobility to allow such compensation; in patients with elbow joint dysfunction, this strategy may not be applicable. Furthermore, since the wrist brace applies the tensile force in this setup, participants who were sensitive to skin deformation reported discomfort on the wrist. To mitigate this issue, future designs should consider replacing the commercial wrist brace with a brace that supports the entire lower arm, reducing localized tensile force and improving user comfort.

Despite promising outcomes, this study has several limitations. First, a diverse patient population with varying stages and severities of FS was not fully represented. For instance, the negative control group also showed noticeable improvement in ROM and SPADI, which may reflect the natural course of recovery in early-stage or mild patients. It is likely that the robotic intervention would exhibit even greater efficacy in more advanced or frozen stages of FS, warranting further investigation. Second, the intervention group received sessions twice per week, whereas the active and negative control groups received therapy once per week. This difference in treatment frequency reflects the practical constraints of therapist availability in clinical settings, while the robotic device allowed for more frequent sessions due to its ease of use and independence from therapist time. Although this may have contributed to the observed therapeutic effects, it also highlights one of the potential advantages of robotic rehabilitation. Future studies could explore the device’s effectiveness across various frequencies and settings, including home-based use, to further validate its clinical utility. Third, while the current device was evaluated in a hospital environment, future studies should explore its feasibility and effectiveness in home-based settings. Specifically, whether patients can operate the device independently and whether its automated monitoring functions can enhance exercise adherence and motivation should be validated. Finally, this study was designed as an exploratory pilot trial with a small sample size, which limits the statistical power and generalizability of the findings. While the results provide preliminary evidence for the feasibility and potential efficacy of the robotic CPM device, future large-scale, adequately powered randomized controlled trials are needed to confirm these findings.

In conclusion, the proposed robotic device for frozen shoulder treatment demonstrated therapeutic effects comparable to manual therapy and superior to thermotherapy. With improved accessibility and reduced dependence on human therapists, the device has significant clinical potential for widespread application. This approach may reduce the dependence on trained therapists and enable wider accessibility for patients with limited mobility or access to clinics.

## Data Availability

The raw data supporting the conclusions of this article will be made available by the authors, without undue reservation.
