# Correction: Development and validation of a sliding type continuous passive motion automation device for evaluation and rehabilitation of frozen shoulder

**DOI:** 10.3389/fresc.2026.1834238

**Published:** 2026-04-07

**Authors:** Jewoo Lee, Sung-sik Yun, Kyung Rok Oh, Sun Gun Chung, Wonjae Hwang, Keewon Kim, Kyu-jin Cho

**Affiliations:** 1 Department of Mechanical Engineering, Seoul National University, Seoul, Republic of Korea; 2 Department of Rehabilitation Medicine, Seoul National University Hospital, Seoul, Republic of Korea; 3 Department of Rehabilitation Medicine, Seoul National University College of Medicine, Seoul, Republic of Korea

**Keywords:** frozen shoulder, rehabilitation, continuous passive motion (CPM), joint stiffness, robot

**Incorrect funding**

Missing

The funder [*Translational Research Program for Rehabilitation Robots grant funded by National Rehabilitation Center, Ministry of Health and Welfare, Korea (#NRCTR-EX24002).*] was erroneously omitted.

The original version of this article has been updated.

In the published article, there was a mistake in the Grant number. The Grant number was displayed as “This work was supported by the National Research Foundation of Korea(NRF) grant funded by the Korea government (MSIT). (No. 2022R1C1C1006057 and RS-2023-00208052)”. The correct Grant number is “This work was supported by the National Research Foundation of Korea(NRF) grant funded by the Korea government (MSIT) (No. 2022R1C1C1006057 and RS-2023-00208052) and Translational Research Program for Rehabilitation Robots grant funded by National Rehabilitation Center, Ministry of Health and Welfare, Korea (#NRCTR-EX24002).”

