# Supplementary material for: Development and validation of a sliding type continuous passive motion automation device for evaluation and rehabilitation of frozen shoulder: a pilot study
Source: Front Rehabil Sci. 2025 Aug 11;6:1639249. doi: 10.3389/fresc.2025.1639249 (PMC12375683; doi:10.3389/fresc.2025.1639249)
Supplement: Supplementary file 1 [file Datasheet1.pdf]

Supplementary Data

Title: Development and Validation of a Sliding Type Continuous Passive Motion Automation Device for Evaluation and Rehabilitation of Frozen Shoulder: A Pilot Study

Software: SAS statistical software (version 9.4; SAS Institute inc., Cary, NC)

Statistical Analysis Result:

This study aimed to compare the outcome measures assessed at three time points—before the intervention, immediately after, and six weeks later—across three groups of patients with adhesive capsulitis (frozen shoulder): the intervention group, which received robotic rehabilitation therapy; negative control group, which received heat-pack therapy; and active control group, which received exercise therapy. Data were obtained from 5 participants in the intervention group, 3 in negative control group, and 4 in active control group.

The demographic characteristics of the participants were analyzed as follows. To evaluate whether there were differences in distributions among the three groups, a non-parametric Kruskal-Wallis test was performed.

Table 1. Demographic characteristics analysis

		Intervention Group<br>N=5	Negative Control<br>N=3	Active Control<br>N=4	p-value*
sex	F, n(%)	4(80)	3(100)	3(75)	>0.9999
	M, n(%)	1(20)	0(0)	1(25)	
age	mean±std	59.6±15.08	58±7	61.25±11.47	0.9674
	median[min,max]	50[47,81]	61[50,63]	60.5[48,76]	
HT	mean±std	160.26±5.77	152.27±2.25	159.7±2.6	0.0394
	median[min,max]	157.8[156.6,170.4]	152.8[149.8,154.2]	158.75[157.8,163.5]	
WT	mean±std	60.8±10.01	69.27±13.19	56.03±9.37	0.5325
	median[min,max]	55.5[51.2,72.7]	61.7[61.6,84.5]	57.15[45.1,64.7]	

* p-values from the Kruskal-Wallis test

Among the demographic characteristics, only HT showed a significant difference in distribution across the three groups at the 5% significance level.

- **Variable of interest: Primary outcome changes from baseline**

We performed analyses on the efficacy outcome measures collected from the three groups. Since the data were measured at baseline, immediately post-intervention, and six weeks post-intervention, a linear mixed model (LMM) was used to account for the repeated measures within individuals.

To assess whether the changes over time differed among the groups, we constructed a linear mixed model with group, time, and baseline values as fixed effects and participant as a random effect. Initially, an interaction term between group and time was included to evaluate whether the relationship between groups and outcome variables changed over time. As the interaction term was not significant for any variable, the final model was constructed without the interaction term.

For each outcome variable, the distributions of measurements at baseline, immediately post-intervention, six weeks post-intervention, as well as change values (post-baseline and six weeks-baseline), were presented alongside the results of the linear mixed model analysis.

1. Forward Flexion

Table 2-1. Forward Flexion

		Intervention Group N=5	Negative Control N=3	Active Control N=4
FF_baseline	mean±std	130±15.81	130±20	127.5±12.58
	median[min,max]	130[110,150]	130[110,150]	130[110,140]
FF_post intervention	mean±std	141±24.6	140±17.32	150±20
	median[min,max]	140[110,170]	130[130,160]	160[120,160]
FF_6 wks post intervention	mean±std	156±13.42	160±10	145±25.17
	median[min,max]	150[140,170]	160[150,170]	150[110,170]
diff_FF_(post intervention-baseline)	mean±std	11±12.45	10±10	22.5±25
	median[min,max]	20[-5,20]	10[0,20]	25[-10,50]
diff_FF_(6 wks post intervention-baseline)	mean±std	26±11.4	30±17.32	17.5±26.3
	median[min,max]	30[10,40]	20[20,50]	25[-20,40]

Table 2-2. Linear mixed model

	Least Squares Means	95% Lower	95% Upper	p-value*
Intervention Group	18.811	1.398	36.225	0.9926
Negative Control	20.311	-2.162	42.785	
Active Control	19.377	-0.124	38.879	

* p-value for group effect from the linear mixed model (adjusted for time and baseline)

The mean change in the intervention group was 18.811, in control group 1 was 20.311, and in control group 2 was 19.377. At the 5% significance level, there was no statistically significant difference in the change of forward flexion (FF) among the three groups.

2. Abduction

Table 3-1. Abduction

		Intervention Group N=5	Negative Control N=3	Active Control N=4
Abd_baseline	mean±std	102±28.64	100±17.32	110±14.14
	median[min,max]	100[70,140]	90[90,120]	105[100,130]
Abd_post intervention	mean±std	114±23.02	106.67±20.82	122.5±20.62
	median[min,max]	110[80,140]	100[90,130]	120[100,150]
Abd_6 wks post intervention	mean±std	126±28.81	133.33±15.28	127.5±22.17
	median[min,max]	120[90,170]	130[120,150]	130[100,150]
diff_Abd_(post intervention-baseline)	mean±std	12±17.89	6.67±30.55	12.5±22.17
	median[min,max]	10[-10,30]	0[-20,40]	10[-10,40]
diff_Abd_(6 wks post intervention-baseline)	mean±std	24±15.17	33.33±25.17	17.5±27.54
	median[min,max]	30[0,40]	30[10,60]	15[-10,50]

Table 3-2. Linear mixed model

	Least Squares Means	95% Lower	95% Upper	p-value
Intervention Group	16.970	-1.553	35.493	0.9961
Negative Control	18.019	-5.970	42.007	
Active Control	17.773	-3.193	38.740	

At the 5% significance level, there was no statistically significant difference in the change of abduction (Abd) among the three groups.

3. External Rotation

Table 4-1. External Rotation

		Intervention Group N=5	Negative Control N=3	Active Control N=4
ER_baseline	mean±std	46±15.17	60±26.46	37.5±5
	median[min,max]	40[30,70]	50[40,90]	40[30,40]
ER_post intervention	mean±std	71±18.17	56.67±11.55	57.5±12.58
	median[min,max]	65[50,90]	50[50,70]	60[40,70]
ER_6 wks post intervention	mean±std	72±19.24	70±20	62.5±5
	median[min,max]	80[40,90]	70[50,90]	60[60,70]
diff_ER_(post intervention-baseline)	mean±std	25±12.25	-3.33±15.28	20±14.14
	median[min,max]	20[10,40]	0[-20,10]	25[0,30]
diff_ER_(6 wks post intervention - baseline)	mean±std	26±11.4	10±10	25±5.77
	median[min,max]	30[10,40]	10[0,20]	25[20,30]

Table 4-2. Linear mixed model

	Least Squares Means	95% Lower	95% Upper	p-value
Intervention Group	25.301	18.171	32.431	0.0241
Negative Control	7.314	-2.860	17.488	
Active Control	19.763	11.255	28.272	

Table 4-3. Post-hoc Analysis (Bonferroni correction)

		Least Squares Means	95% Lower	95% Upper	p-value	Adj p-value
Active Control	Intervention Group	-5.538	-16.580	5.505	0.3071	0.9212
Active Control	Negative Control	12.450	-1.757	26.656	0.0823	0.247
Intervention Group	Negative Control	17.987	5.488	30.486	0.0072	0.0215

At the 5% significance level, there was a statistically significant difference in the change of external rotation (ER) among the three groups (p-value = 0.0241). Accordingly, pairwise comparisons between groups were performed. As a result, a significant difference was observed between the intervention group and control group 1 (Bonferroni-adjusted p-value = 0.0215 to control for the increased risk of type I error due to multiple comparisons).

- **Variable of interest: Secondary outcome changes from baseline**

1. Pain

Table 5-1. Pain

		Intervention Group N=5	Negative Control N=3	Active Control N=4
pain_baseline	mean±std	55.2±25.95	66±27.71	67.5±29.14
	median[min,max]	58[24,84]	82[34,82]	65[40,100]
pain_post intervention	mean±std	34.4±18.46	34±22.54	43.5±43.68
	median[min,max]	36[12,56]	22[20,60]	37[6,94]
pain_6 wks post intervention	mean±std	29.6±13.74	32.67±15.14	25±32.68
	median[min,max]	30[10,46]	26[22,50]	9[8,74]
diff_pain_(post intervention-baseline)	mean±std	-20.8±25.4	-32±24.58	-24±15.06
	median[min,max]	-22[-56,14]	-22[-60,-14]	-25[-40,-6]
diff_pain_(6 wks post intervention-baseline)	mean±std	-25.6±21.61	-33.33±22.03	-42.5±21.56
	median[min,max]	-28[-52,4]	-32[-56,-12]	-35[-74,-26]

Table 5-2. Linear mixed model

	Least Squares Means	95% Lower	95% Upper	p-value
Intervention Group	-25.642	-43.861	-7.423	0.8682
Negative Control	-31.230	-54.441	-8.019	
Active Control	-31.275	-51.483	-11.067	

At the 5% significance level, there was no statistically significant difference in the change of pain among the three groups.

2. Disability

Table 6-1. Disability

		Intervention Group N=5	Negative Control N=3	Active Control N=4
disability_baseline	mean±std	40.75±29.85	63.33±23.46	69.06±24.78
	median[min,max]	50[8.75,81.25]	76.25[36.25,77.5]	65.63[45,100]
disability_post intervention	mean±std	27.25±14.67	22.08±10.1	32.5±37.6
	median[min,max]	32.5[10,43.75]	23.75[11.25,31.25]	21.88[2.5,83.75]
disability_6 wks post intervention	mean±std	22.25±15.52	23.75±17.37	23.44±34.48
	median[min,max]	16.25[5,45]	15[12.5,43.75]	8.13[2.5,75]
diff_disability_(post intervention-baseline)	mean±std	-13.5±25.79	-41.25±14.42	-36.56±26.23
	median[min,max]	-13.75[-40,18.75]	-46.25[-52.5,-25]	-29.38[-71.25,-16.25]
diff_disability_(6 wks post intervention-baseline)	mean±std	-18.5±23.69	-39.58±22.72	-45.63±21.25
	median[min,max]	-33.75[-36.25,16.25]	-32.5[-65,-21.25]	-41.25[-75,-25]

Table 6-2. Linear mixed model

	Least Squares Means	95% Lower	95% Upper	p-value
Intervention Group	-23.155	-44.679	-1.631	0.6286
Negative Control	-36.859	-62.827	-10.891	
Active Control	-34.818	-58.185	-11.452	

At the 5% significance level, there was no statistically significant difference in the change of disability among the three groups.

3. Total

Table 7-1. Total

		Intervention Group N=5	Negative Control N=3	Active Control N=4
total_baseline	mean±std	46.3±28.05	64.36±25.1	68.46±22.85
	median[min,max]	53.07[14.61,82.3]	78.46[35.38,79.23]	64.23[45.38,100]
total_post intervention	mean±std	30±15.8	26.66±14.19	36.73±39.6
	median[min,max]	36.15[13.07,48.46]	23.07[14.61,42.3]	27.69[3.84,87.69]
total_6 wks post intervention	mean±std	25.05±14.65	27.18±16.43	24.29±34.27
	median[min,max]	20.7[6.92,45.38]	17.69[17.69,46.15]	8.46[4.61,75.61]
diff_total_(post intervention-baseline)	mean±std	-16.31±25.11	-37.7±17.32	-31.73±20.73
	median[min,max]	-16.92[-46.16,16.93]	-36.93[-55.39,-20.77]	-29.23[-56.15,-12.31]
diff_total_(6 wks post intervention-baseline)	mean±std	-21.26±22.39	-37.18±22.33	-44.17±16.13
	median[min,max]	-32.37[-40.77,11.47]	-32.31[-61.54,-17.69]	-46.92[-58.46,-24.39]

Table 7-2. Linear mixed model

	Least Squares Means	95% Lower	95% Upper	p-value
Intervention Group	-23.319	-43.035	-3.604	0.6423
Negative Control	-35.090	-59.385	-10.796	
Active Control	-34.039	-55.603	-12.475	

At the 5% significance level, there was no statistically significant difference in the change of total among the three groups.
